# Intergenerational effects of dietary iron on swimming and metabolic performance in zebrafish

**DOI:** 10.3389/fphys.2025.1693900

**Published:** 2025-10-16

**Authors:** Theanuga Chandrapalan, Suhani Walia, Raymond W. M. Kwong

**Affiliations:** Department of Biology, York University, Toronto, ON, Canada

**Keywords:** dietary iron, metal homeostasis, respirometry, swimming, reproduction, intergenerational effects, zebrafish

## Abstract

Iron is an essential trace metal required for various physiological processes, yet both deficiency and excess can disrupt metal homeostasis and compromise fitness. In this study, we investigated how dietary iron availability influences physiological performance across generations in zebrafish (*Danio rerio*). Fish were fed diets spanning a gradient from deficiency to supplementation (Low Fe, 11 mg Fe/kg; Medium Fe, 420 mg Fe/kg, and High Fe, 2,300 mg Fe/kg), and effects on growth, metal homeostasis, swimming performance, energy metabolism, and reproduction were assessed. Following reproductive assays, offspring were raised under control conditions and subsequently challenged with the same dietary iron treatments (Low Fe, Medium Fe, and High Fe as parents) in adulthood. Sub-acute exposure (20 days) to elevated dietary iron enhanced aerobic scope, maximum metabolic rate, and critical swimming speed, alongside improved reproductive output as measured by embryo survival and early development. However, sub-chronic exposure (40 days) to High Fe diminished swimming performance benefits and was also associated with tissue iron loading. Notably, zebrafish tolerated sub-chronic exposure to Low Fe without significant impacts on condition factor or energetic performance. Interestingly, the difference in swimming and metabolic performance between high and low iron treatments was more pronounced in the offspring, suggesting an intergenerational effect of parental iron status. Together, these findings suggest that dietary iron availability can shape both immediate and inherited performance phenotypes, underscoring its dual role as a nutritional requirement and a regulator of ecological fitness.

## 1 Introduction

Iron is an essential micronutrient for all fish; it is a vital component of a multitude of protein complexes, a cofactor for many enzymes, and is involved in various biochemical and metabolic processes such as oxygen transport, energy metabolism, and development (Wood et al., 2011; Chandrapalan and Kwong, 2021). However, imbalances in iron levels, both deficiency and overload, can negatively impact fish health, leading to physiological impairments such as reduced growth, disrupted oxygen transport, oxidative stress, and metal accumulation (Watanabe et al., 1997; Lall and Kaushik, 2021). In freshwater environments, iron concentrations are usually low (<1 mg/L), but hypoxia, acidification, and anthropogenic activities (mining and industrial discharge) can substantially elevate levels and increase availability (Peuranen et al., 1994; Xing and Liu, 2011; Kwong, 2024). Within physiologically safe concentrations, iron supplementation can be important for the maintenance of fish health, including growth, feed utilization, and immune function (Wang et al., 2023). While diet is considered the predominant route of iron acquisition, the effects of dietary iron exposure have been far less studied than waterborne uptake, leaving critical gaps in our understanding of how dietary iron availability impacts fish physiological performance (Andersen, 1997; Bury, 2003; Jee et al., 2004; Lappivaara and Marttinen, 2005; Wang, 2013; Saha et al., 2015). In general, dietary iron overload is linked to iron loading in tissues, which can catalyze the Fenton reaction and the production of reactive oxygen species and oxidative stress, whereas dietary iron deficiency impairs hemoglobin synthesis, oxygen transport capacity, and reduces energetic performance in fish (Gallaugher et al., 1995; Andersen et al., 1996; Galbraith et al., 2019). However, no study has comprehensively examined how dietary iron deficiency and supplementation influence key survival traits such as sustained swimming performance, metabolic capacity, and reproductive success, and whether such effects can extend to subsequent generations.

Swimming performance is an integrative measure of physiological capacity because it incorporates oxygen transport, energy metabolism, and muscle function (Martínez et al., 2003; Rubio-Gracia et al., 2020). Ecologically, swimming ability is crucial for avoiding predation, foraging for food, migration, and mating success, making it a key determinant of fitness (Tudorache et al., 2008; Cano-Barbacil et al., 2020). Consequently, swimming assays are widely used as sensitive biomarkers of environmental and nutritional stress (Little and Finger, 1990). Previous studies on other metals or metalloids have demonstrated that exposure to elevated levels of copper, selenium, and cadmium can impair swimming ability and alter metabolic rates in multiple species (minnows, trout, and sturgeon) (Massé et al., 2013; McPhee and Janz, 2014; Cunningham and McGeer, 2016; Puglis et al., 2019). In contrast, one study on iron supplementation (∼1500 mg Fe/kg) reported improved burst swimming activity in masu salmon (*Oncorhynchus masou*) (Mizuno et al., 2007). Nonetheless, sustained swimming activities are energetically costly and are underpinned by metabolic capacity. Fish partition energy among basal maintenance, locomotion, and reproduction, and this balance is strongly influenced by nutrient availability (Ohlberger et al., 2006; Mcbride et al., 2015; Chabot et al., 2016). Since iron is directly involved in mitochondrial respiration and enzymatic activities, dietary iron levels have the potential to affect metabolic rates and energy balance (Judge and Dodd, 2020; Chandrapalan and Kwong, 2021). However, whether dietary iron deficiency or supplementation can also alter swimming performance remains largely unexplored.

Beyond swimming and metabolic performance, iron exposure may also affect reproductive success and influence intergenerational fitness. Intergenerational effects occur when parental environments shape offspring traits via altered nutrient provisioning, direct metal transfer to gametes, or epigenetic reprogramming (Thomas and Janz, 2014; Beaver et al., 2017; Domínguez-Petit et al., 2022). Such effects are increasingly recognized as critical for understanding population responses to environmental stressors, including metals. In fish, parental exposure to metals has been linked to metal accumulation in reproductive tissues and altered reproductive performance, including fecundity, egg quality, and hatching success (Taslima et al., 2022). Most oviparous fish, like zebrafish (*Danio rerio*), rely on maternally supplied yolk nutrients during early development and can be particularly vulnerable to these parental influences (Beaver et al., 2017; Thomason et al., 2017). Moreover, swimming performance in zebrafish has been shown to have a heritable component, and parental metal exposure was linked to persistent changes in offspring swimming ability and metabolism (Thomas and Janz, 2015; Wakamatsu et al., 2019). Investigating whether dietary iron availability affects reproductive performance and shapes offspring physiology is therefore essential to evaluate the ecological and intergenerational consequences of micronutrient stress.

In addition, iron exposure may have broader implications beyond its direct physiological roles, as iron is known to compete with or facilitate the uptake of other trace metals and major ions. Consequently, dietary iron deficiency or supplementation may disrupt systemic trace metal and ion homeostasis, with effects extending beyond iron regulation alone. These interactions between metals and metal transport pathways are further reflected in tissue-specific responses. For example, the brain is a highly metabolically active site and is sensitive to iron-induced oxidative stress; the intestine is the primary site of dietary metal absorption; the liver functions as the central hub for iron storage and metabolic regulation; and the ovaries represent a direct link between metal bioaccumulation to reproductive success, and potential maternal transfer (Kwong and Niyogi, 2008; Anderson and Shah, 2013; Thomas and Janz, 2015; Hassan and Kwong, 2020). Thus, understanding how dietary iron exposure affects the accumulation of metals and ions across these key tissues provides crucial insight into the mechanistic pathways by which iron may shape physiological performance and reproductive outcomes.

In the present study, we tested the hypothesis that dietary iron levels influence swimming performance by altering metabolic capacity and energy use, and affect reproductive success. We further hypothesized that these effects extend across generations and alter offspring performance. To address these questions, the freshwater teleost, zebrafish, with well-characterized physiology, tractability for dietary manipulation, and validated assays for swimming, metabolism, and reproduction, was chosen as the model species. Adult zebrafish were fed an iron-deficient (11 mg Fe/kg; Low Fe), adequate (420 mg Fe/kg; Medium Fe), or iron-enriched (2,300 mg Fe/kg; High Fe) diet for 20 or 40 days. The concentrations of iron in the Low Fe, Medium Fe, and High Fe diets were selected to reflect fluctuating iron levels in the environment that span a biologically relevant gradient from deficiency, to adequacy, to supplementation. The Medium Fe diet was considered nutritionally adequate as it meets the baseline recommended levels in aquaculture (∼30–170 mg Fe/kg) while also closely resembling the composition of commercial zebrafish diet (e.g., Zeigler) commonly used in laboratory rearing (Watanabe et al., 1997). To capture both early and longer-term responses, we examined 20-day (sub-acute) and 40-day (sub-chronic) exposures. These durations align with toxicological testing windows (∼14–30 days), typical zebrafish nutritional trials (∼3–10 weeks), and are ecologically relevant given the species’ rapid life cycle and sexual maturation at ∼3 months (Cooper et al., 2006; Singleman and Holtzman, 2014; Beaver et al., 2017; Chandrapalan and Kwong, 2020). We assessed: i) trace metal and major ion accumulation in the brain, intestine, liver, and ovaries, ii) swimming and metabolic performance, and iii) reproductive output. To evaluate potential intergenerational effects, the offspring of iron-exposed parent fish were raised under control conditions to adulthood before being challenged with the same dietary iron treatments as their parents. By integrating organ-specific metal analyses with performance-based assessments across two generations, this study provides novel insight into the role of dietary iron in regulating metabolism, reproduction, and intergenerational fitness in fish.

## 2 Materials and methods

### 2.1 Animals and experimental design

Adult zebrafish (Tüpfel long-fin strain) were housed in recirculating systems (Aquaneering, CA, USA) and maintained at 28 °C with a 14 h light: 10 h dark photoperiod at York University’s zebrafish vivarium. They were fed brine shrimp in the morning and commercial zebrafish pellets to satiation in the afternoon (Zeigler, PA, USA; comprised of 55% protein; 15% fat; 1.5% fiber; 12% ash; 1.5% phosphorus; 2.78% calcium; and 0.37% sodium). During weekends, they were fed once a day with pellets. The measured trace metal content in the Zeigler diet was 309.00 mg Fe/kg, 208.00 mg Zn/kg, 57.00 mg Mn/kg, 40 mg Cu/kg, 0.41 mg Co/kg, and 1.19 mg I/kg. All animal work was conducted in accordance with the Canadian Council for Animal Care and approved by the York University Animal Care Committee (2017–2 R2).

**FIGURE 1:**
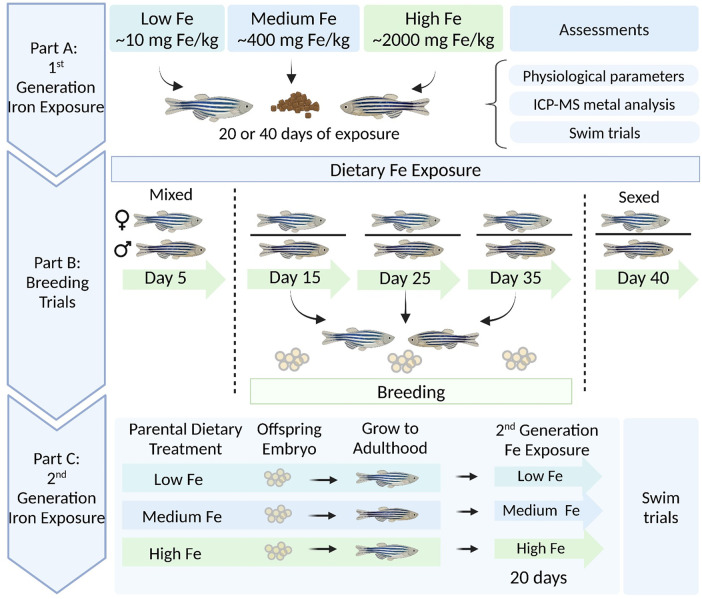
Schematic overview of dietary (parent and offspring) iron exposure, breeding trials, and intergenerational swim performance trials in zebrafish. Part **(A)** Adult zebrafish were exposed to three experimental diets differing in iron content (Low Fe, Medium Fe, and High Fe) for 20 or 40 days. Following exposure, fish were assessed for physiological parameters, tissue metal accumulation, and swim performance. Part **(B)** Concurrent breeding trials were conducted at 10-day intervals between days 5 and 40 of exposure. Fertilized embryos were collected from each dietary treatment group. Part **(C)** Offspring from Day 15 breeding session were raised to adulthood under standard conditions, then exposed for 20 days to the same dietary iron treatment as their parents. Swim performance was examined in parent and offspring generations to assess intergenerational effects of dietary iron exposure.

A total of 150 adult zebrafish (∼12 months of age) were used in this study, and 25 fish were allocated per treatment group. Each group of 25 was split into two tanks (2.8 L) with an approximately equal ratio of males to females (12–13 fish per tank). In brief, the fish were subjected to a feeding trial (Section 2.2), physiological assessments (Section 2.3), and respirometry trials (Section 2.4). Throughout the dietary iron exposure, the fish also participated in repeated breeding trials (Section 2.5), and offspring from Day 15 were used to assess the potential intergenerational effects of parental dietary exposure. A schematic illustrating the experimental design is shown in Figure 1.

### 2.2 Preparation of experimental diets and dietary treatment

Three experimental diets ranging in iron content were prepared as described previously (Kwong et al., 2013; Chandrapalan and Kwong, 2020), with some modifications. A purified low-iron zebrafish diet (Dyets Inc; nutritional composition listed in Supplementary Table S1) was formulated by replacing high-iron protein sources (i.e., casein, wheat gluten, and egg white) with purified L-amino acids and ingredients lower in iron content (i.e., microcrystalline cellulose). Inductively coupled plasma mass spectrometry (ICP-MS) analysis (Water Quality Center, Trent University) determined the iron concentration in this purified diet to be 7.5 ± 0.2 mg Fe/kg (mean ± SEM, n = 4). To prepare the Medium and High Fe diets, an appropriate amount of iron (as $\text{FeS}O_{4}\cdot 7H_{2}O$ ) was dissolved in deionized water and mixed with the purified diet. The resulting paste was dehydrated at 65 °C for 2 days. The food was then portioned into edible sizes (∼1 mm) and stored at 4 °C until use. The Low Fe diet was prepared in the same manner but without the addition of FeSO_4_. The final iron concentrations in the three diets were 11 ± 1 (Low Fe), 419 ± 46 (Medium Fe), and 2,280 ± 75 (High Fe) mg Fe/kg (Supplementary Table S2). The elemental composition of the purified diet and experimental diets are reported in Supplementary Table S3. Among the three dietary iron levels, the Medium Fe diet was considered the control, as its iron content was similar to that of the commercial zebrafish diet (i.e., Zeigler) used for routine zebrafish rearing alongside brine shrimp supplementation, although brine shrimp were not provided during the experimental feeding period.

At the start of the feeding trials, all fish were acclimated to the Medium Fe diet for 1 week to ensure adjustment to the new feeding regime and experimental diet. Thereafter, the fish were divided into six groups: one group of three received a 20-day dietary exposure, while the other three groups received a 40-day exposure. The fish were fed the Low, Medium, or High Fe diets twice daily (totaling 5% of body weight) on weekdays and once daily on weekends. During feeding, the recirculating system (Aquaneering, CA, USA) was turned off, and the water in each tank was continuously flushed for 1 hour. To assess potential iron leaching from the diet, water samples were collected after feeding, and ICP-MS analysis indicated negligible leaching, with measured iron concentration of 2.03 ± 0.85 µg Fe/L (*n* = 4). The iron concentration in the water of the recirculating system was approximately 1.2 µg Fe/L.

### 2.3 Physiological parameters, tissue metal loading, and energy reserves

Physiological parameters such as standard body length (SL; the distance from the tip of the snout to the base of the caudal peduncle) (cm), wet weight (g), and condition factor [100 x weight (g)/length^3^ (cm)] were measured at the start and end of all feeding trials (Day 20 or 40). Hepatosomatic index [HSI; 100 x (liver weight (g)/body weight (g)] and gonadosomatic index [GSI; 100 x (gonad weight (g)/body weight (g)] were measured at the completion of feeding trials. The fish were fasted for 24 h to clear the gut contents prior to euthanasia with an overdose of tricaine methanesulfonate (MS-222) buffered with sodium bicarbonate to pH 7 (0.4 g/L MS-222) and tissue collection. Brain, intestine, liver, and ovaries were collected, and organs from three to five fish from each experimental group were pooled to form one replicate. A total of three to four replicates were collected for each organ, and all tissue samples were dehydrated at 65 °C overnight and then digested with 6N HNO_3_ for 48 h. The samples were diluted in 2% HNO_3_, filtered (0.45 µm), and analyzed for metal concentrations using ICP-MS (Agilent, 8800 ICP-QQQ-MS, CA, USA) at the Water Quality Centre, Trent University (ON, CA). NIST SRM 1640a (Trace Elements in Natural Water) was used for QA/QC, and the measured concentrations were within 5% of the certified values.

Following organ extraction, fish carcasses were weighed, flash-frozen in liquid nitrogen, and stored at −80 °C until use. Glycogen and triglyceride, which are primary energy sources utilized during burst and sustained swimming activities, were quantified in the carcass using colorimetric kits from Abcam following the manufacturer’s instructions (Ab169558 and Ab65336).

### 2.4 Swimming and metabolic performance

Two Loligo miniswim tunnel respirometers (Loligo® Systems, Viborg, Denmark) with 170 mL swim tunnels were used for all intermittent-flow respirometry trials. Each Loligo system was connected to a recirculating system with a flush pump (Eheim Universal 1046 pump) and externally mixed to ensure homogenous oxygen distribution. Oxygen and velocity calibrations were conducted before the onset of the swim trials. The external water bath with a submersible aquarium heater was used to maintain a constant temperature (28 °C), and both chambers were visually isolated using opaque shielding to minimize external disturbances and reduce stress to fish. The lighting conditions were maintained constant throughout the experiment, and all trials were conducted during the daytime.

Oxygen saturation (%) was monitored continuously using oxygen probes (Dipping probe mini sensor, Loligo® Systems) connected to an oxygen meter (Witrox 4, Loligo® Systems). Readings were recorded at a frequency of approximately 1 Hz, and saturation was maintained above 85%. Chamber temperature was maintained at 28 °C ± 0.5 °C and monitored with a temperature probe (Temperature sensor Pt1000, Loligo® Systems) connected to the Witrox 4 system. AutoResp™ 2 software (Loligo® Systems) was used to automate the intermittent-flow respirometry cycles and measure oxygen consumption. Each cycle consisted of a flush phase (1min 30s), a wait phase (30s), and a measurement phase (3min).

For critical swimming speed (Ucrit) tests (Supplementary Figure S1), fish were transferred into the Loligo swim tunnel (inner swim chamber) quickly to minimize handling stress and allowed to acclimate to the new environment for 30 min at a velocity of 3.5 cm/s, or until their oxygen consumption rates stabilized. After 30 min, the critical swim test began, during which the water velocity was increased by 3.5 cm/s every 5 min until the fish reached fatigue, defined as consistent failure to swim against the current and resting against the back of the swim tunnel. Once the swim test concluded, the velocity was reset to the acclimation speed to allow the fish to rest briefly (∼5 min) before being removed from the swim tunnel. The fish were then sacrificed to measure physiological indices and collect tissue for metal analysis and energy storage assays (as described above). Oxygen consumption (MO_2_), cost of transport (COT), standard metabolic rate (SMR), maximum metabolic rate (MMR), aerobic scope (AS), and U_crit_ (body lengths/second, BL/s; the average length of the fish was 3.2 cm) were determined. SMR was calculated using linear regression analysis to extrapolate MO_2_ values to 0 swimming speed. COT was calculated by multiplying MO_2_ by an oxycaloric value of 14.1 J/mg O_2_ and then dividing by the corresponding swimming speed (m/s) (Videler, 1993). U_crit_ was calculated as follows:

$$U_{crit}=U_{f}+\left(T_{f}/T_{i}\right)\times U_{i}$$

U_f_ is the highest velocity (cm/s) maintained by the fish for a whole interval (5 min). T_f_ is the time elapsed at fatigue (min), and T_i_ is the prescribed time interval (min). U_i_ is the prescribed velocity increment (cm/s). U_crit_ was corrected for the body length of the fish (BL/s).

### 2.5 Reproductive capacity and subsequent responses of the offspring to iron challenges

The breeding capacity of zebrafish was examined at 15, 25, and 35 days of dietary iron treatment. Before the onset of breeding trials, all fish were pre-bred once and maintained in mixed-sex groups to allow embryo release. At day 5 of iron treatment, males and females were separated, and this separation was maintained for 10 days between each breeding session on days 15, 25, and 35. This timeline ensured that both sexes were isolated for an equal duration (10 days) between breeding sessions. Eight breeding pairs were established for each dietary iron treatment group (Low Fe, Medium Fe, and High Fe), consisting of one male and one female per pair. Breeding pairs were set up in separate breeding tanks overnight and bred in the morning. Following breeding, the sexes were returned to their original tanks. All embryos were collected and transferred to 50 mL Petri dishes (20 embryos per dish) to quantify total embryo production. Eighty embryos (20 embryos per replicate, *n* = 4 per treatment) were collected for ICP-MS analysis (as described previously for tissue samples; section 2.3). Survival rate at 1 day post-fertilization (dpf) for each breeding session (Day 15; *n* = 28–29, Day 25; *n* = 6–32, and Day 35; *n* = 3–12) (each replicate consists of 20 embryos) and standard body length at 5 dpf were measured (*n* = 4–10). All remaining embryos were euthanized, except those from the Day 15 breeding session, which were raised into adulthood for the next part of the study (intergenerational iron exposure).

To assess how the offspring of iron-exposed parents (F_1_ generation) responded to dietary iron treatment compared to their parents (F_0_), embryos from the Day 15 breeding session were raised to adulthood (under standard dietary regimes; Section 2.1) and then re-exposed to the same experimental conditions (Low Fe, Medium Fe, or High Fe; Section 2.2) as their parents for 20 days. The feeding and respirometry trials were conducted using the same protocols as for the parent generation (as outlined above). Physiological parameters (SL, weight, and condition factor) were measured at the start and end of the dietary iron exposure. All fish were fasted for 24 h and subjected to the critical swim test.

### 2.6 Statistical analysis

Statistical analyses were performed using R (Version 4.1.3). Normality and homogeneity of data were assessed using the Shapiro-Wilk and Brown-Forsythe tests prior to all parametric tests. Two-way analysis of variance (ANOVA) or two-way repeated measures ANOVA followed by a Holm-Sidak multiple comparisons test was utilized to determine any statistical significance (*p* < 0.05) of the dietary iron treatment and duration of iron exposure on multiple physiological parameters (tissue metal accumulation, swimming performance, and offspring development). One-way ANOVA followed by a Holm-Sidak multiple comparisons test was utilized to determine any statistical significance (*p* < 0.05) within dietary treatments or exposure duration (physiological parameters of parents, offspring swim performance, and fatigue measurements). Permutation-based two-way ANOVA (10,000 iterations), followed by pairwise permutation tests (FDR-adjusted, *p* < 0.05), was used when assumptions were not met to compare group differences (average embryo survival). All graphs were constructed in R using the package ggplot2. Boxplots show the mean (central yellow diamond), median (horizontal line), upper and lower quartiles, and 1.5× interquartile range.

## 3 Results

### 3.1 Physiological indices

The effects of three different diets with low (11 mg Fe/kg), medium (420 mg Fe/kg), and high (2,300 mg Fe/kg) levels of iron were assessed over two exposure periods: 20 days and 40 days. Physiological parameters, including condition factor, HSI, and GSI, were analysed separately for each sex (Table 1). Following both 20- and 40-day exposures, there were no statistically significant differences in various physiological indices (condition factor, HSI (%), and GSI (%)) among the dietary iron treatment groups. Additionally, dietary iron treatments did not affect zebrafish survival as only one mortality was recorded, occurring on Day 34 in the Low Fe treatment group (Day 40 trial; data not shown).

**TABLE 1:** Condition factor (g/cm^3^), hepatosomatic index (HSI), and gonadosomatic index (GSI) of male and female zebrafish exposed to different dietary iron treatments (Low, Medium, and High Fe) for 20 or 40 days. Lowercase letters denote statistically significant differences between exposure durations within each dietary treatment and sex (Student’s t-test, p < 0.05). Data are mean ± SEM; n = 9–13.

Sex	Diet	Condition factor		HSI (%)		GSI (%)	
		Day 20	Day 40	Day 20	Day 40	Day 20	Day 40
Male	Low Fe	1.7 ± 0.05^a^	1.4 ± 0.09^b^	0.84 ± 0.09^a^	0.59 ± 0.09^b^	0.64 ± 0.09^a^	0.31 ± 0.07^b^
	Medium Fe	1.6 ± 0.06^a^	1.3 ± 0.07^b^	0.91 ± 0.09^a^	0.71 ± 0.1^b^	0.77 ± 0.2^a^	0.31 ± 0.06^b^
	High Fe	1.8 ± 0.07^a^	1.4 ± 0.04^b^	1.3 ± 0.2^a^	0.84 ± 0.1^b^	0.69 ± 0.09^a^	0.56 ± 0.1^a^
Female	Low Fe	1.7 ± 0.05^a^	1.7 ± 0.07^a^	1.9 ± 0.2^a^	1.2 ± 0.1^a^	8.1 ± 1^a^	2.5 ± 0.4^b^
	Medium Fe	1.8 ± 0.08^a^	1.6 ± 0.08^a^	1.4 ± 0.1^a^	1.1 ± 0.1^a^	5.6 ± 1^a^	3.6 ± 0.6^a^
	High Fe	1.9 ± 0.06^a^	1.6 ± 0.06^b^	1.9 ± 0.3^a^	1.4 ± 0.1^b^	8.2 ± 1^a^	3.5 ± 0.4^b^

However, there were significant differences between the duration of iron exposure (Day 20 and Day 40) within each diet (Table 1). Specifically, condition factor, HSI, and GSI were generally higher in fish from the Day 20 group compared to those from the Day 40 group, across all treatments in males except for GSI within the High Fe group. In females, condition factor and HSI were stable in Low and Medium Fe groups, but not in the High Fe group, and the GSI of the Low Fe group. Only females in the Medium Fe group maintained condition factor, HSI, and GSI at both sampling time points.

Consistent with the generally higher physiological indices observed in the Day 20 group, the body weight of fish used in the Day 20 experiment was also higher than that of the Day 40 group, both pre- and post-exposure (Supplementary Table S4). Likewise, concurrent and repeated breeding trials may have resulted in temporal variation, as reflected by significantly lower GSI values within the Day 40 groups.

### 3.2 Tissue burden of trace metals and major ions

**FIGURE 2:**
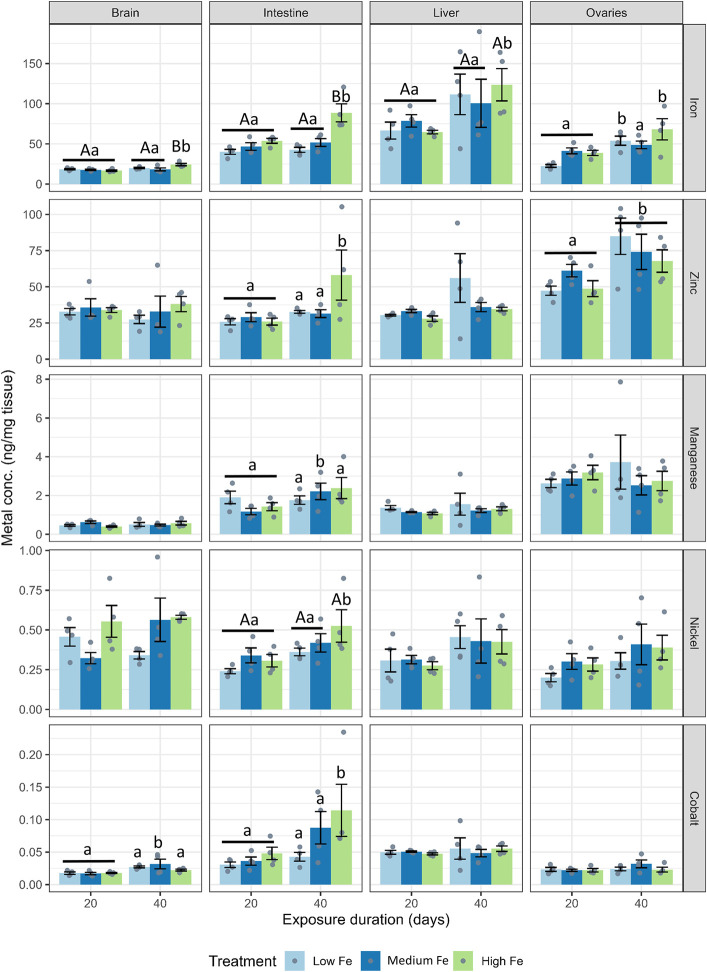
Tissue-specific accumulation of iron and other divalent metals in zebrafish following dietary iron exposure. Concentrations of iron, zinc, manganese, nickel, and cobalt (ng/mg tissue) were measured in the brain, intestine, liver, and ovaries after 20 or 40 days of exposure to Low Fe, Medium Fe, or High Fe diets. Data are mean ± SEM; n = 3–4 (each replicate consisted of a pooled tissue sample from three to five fish). Differing uppercase letters denote statistically significant differences among dietary iron treatments within an exposure timepoint. Differing lowercase letters indicate differences between exposure durations within the same dietary iron treatment. Two-way ANOVA followed by a *post hoc* Holm–Sidak test; *p* < 0.05.

Tissue samples from the brain, intestine, liver, and ovaries were collected for trace metal and major ion analysis. A bar chart showing trace metal concentrations across tissues and dietary iron treatments (Low, Medium, High Fe) is presented in Figure 2, with summarized heatmaps in Supplementary Figure S2. The liver consistently exhibited the highest iron concentrations across all dietary treatments. Iron levels in various organs were influenced by both dietary iron levels and the exposure duration. Specifically, intestinal iron concentrations in the High Fe group increased significantly from Day 20 to Day 40 (*p* < 0.001). Significant increases in iron accumulation were also observed in the brain (*p* < 0.001), liver (*p* < 0.05), and ovaries (*p* < 0.01) after 40 days of High Fe exposure.

Zinc concentrations in the ovaries also increased at Day 40, but this effect was independent of dietary treatment (Figure 2; Supplementary Figure S2). At Day 40, an increase in intestinal zinc levels was also observed in fish from the High Fe group (*p* < 0.01). Manganese, nickel, and cobalt levels in the liver and ovaries were largely unaffected by dietary iron levels. However, nickel and cobalt concentrations in the intestine increased between Day 20 and Day 40 in the High Fe group. Within the Medium Fe group, manganese and cobalt showed elevated levels in the intestine and brain, respectively. Copper levels were measured but showed no significant treatment effects (data not shown).

**FIGURE 3:**
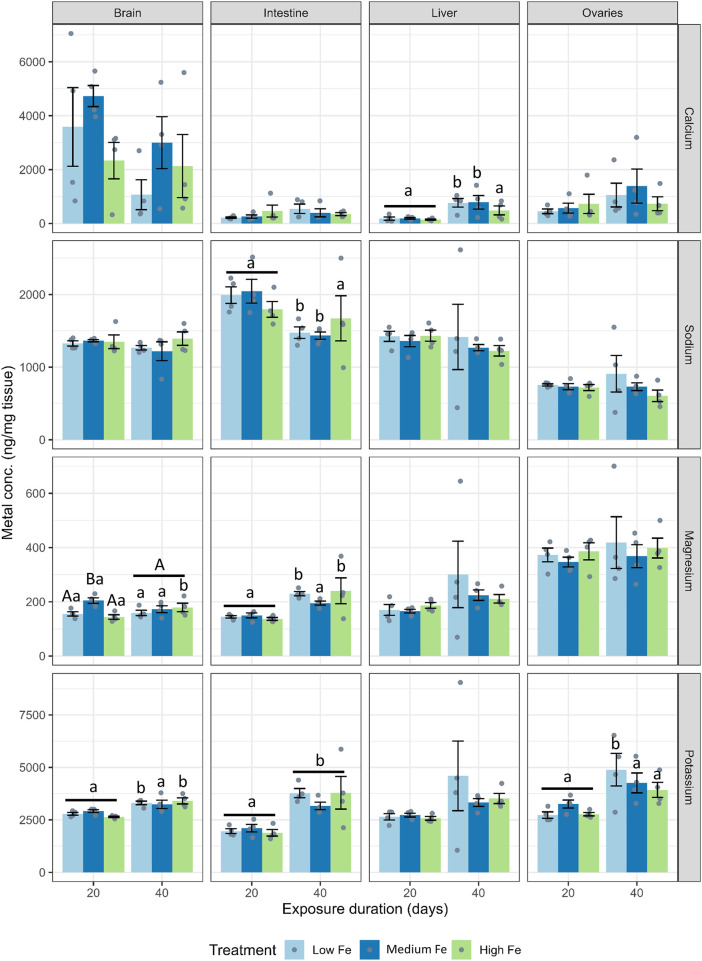
Effects of dietary iron exposure on concentrations of major ions in zebrafish tissues. Calcium, sodium, potassium, and magnesium concentrations (ng/mg tissue) were measured in the brain, intestine, liver, and ovaries after 20 or 40 days of exposure to Low Fe, Medium Fe, and High Fe diets. Data are mean ± SEM; n = 3–4 (each replicate consisted of a pooled sample from three to five fish). Differing uppercase letters indicate statistically significant differences among dietary iron treatments within an exposure duration. Lowercase letters indicate differences between exposure durations within the same dietary iron treatment. Two-way ANOVA followed by a *post hoc* Holm–Sidak test; *p* < 0.05.

For major ions, dietary iron treatments did not have significant effects across most organs (Figure 3; Supplementary Figure S2). Instead, major ion levels appeared to fluctuate from Day 20 to Day 40 within dietary Fe treatments. At Day 40, hepatic calcium levels were higher in fish fed the low and medium Fe diet when compared with fish fed the high Fe diet (*p* < 0.01 in both dietary iron groups).

### 3.3 Swimming capacity and energy metabolism

**FIGURE 4:**
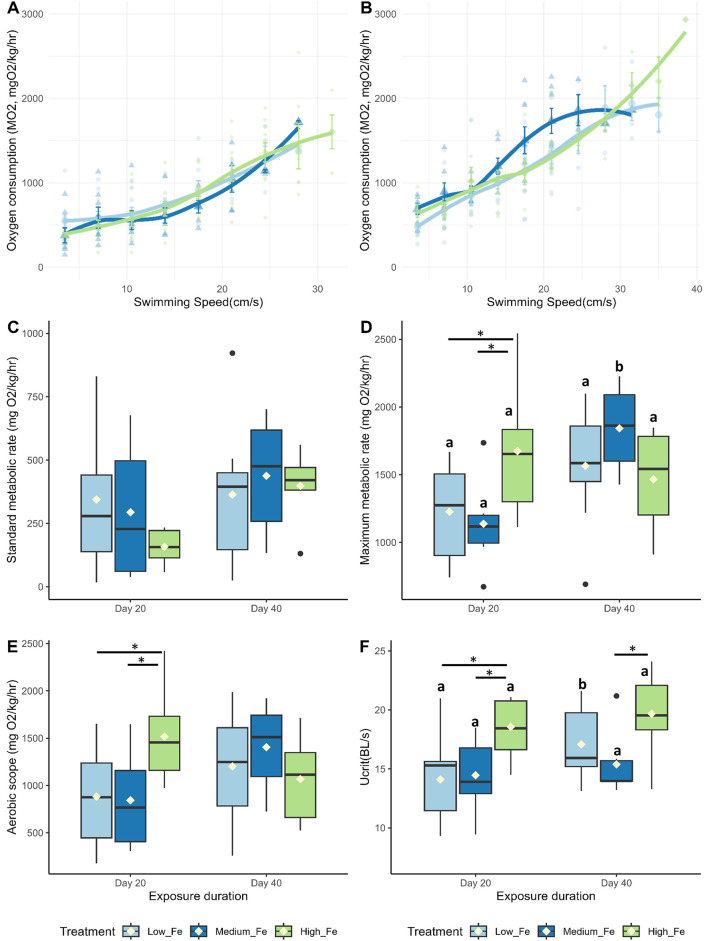
Effects of dietary iron exposure on swimming performance and metabolic rates of adult zebrafish exposed to dietary iron. Oxygen consumption rates (MO_2_, mgO_2_/kg/hr) during a critical swim test following **(A)** 20 or **(B)** 40 days of Low Fe, Medium Fe, and High Fe treatment. Data are mean ± SEM; n = 7–12 fish at starting speeds and n = one to three fish at ending speeds. Each data point represents an individual measurement, with different symbols/colors indicating different dietary iron groups. Non-linear regression lines were fitted using locally estimated scatterplot smoothing (LOESS) to illustrate trends in metabolic demand across swimming speeds. Statistical differences were assessed using a two-way repeated-measures ANOVA, followed by *post hoc* Holm-Sidak test, *p* < 0.05. **(C)** Standard metabolic rate, **(D)** maximum metabolic rate, **(E)** critical swimming speed, and **(F)** aerobic scope of adult zebrafish exposed to dietary iron treatment for 20 or 40 days. Asterisks denote statistical significance (^*^ *p* < 0.05) among dietary iron treatments (Low, Medium, and High Fe) within exposure duration, and lowercase letters denote significant differences between exposure durations (Day 20 vs. Day 40) within the same dietary iron treatment. Two-way ANOVA; *p* < 0.05, n = 7-9 fish.

On both Day 20 and Day 40, the High Fe groups consistently outperformed the other two dietary treatment groups in terms of swimming endurance during the critical swim test, often lasting one to three cycles longer (Figures 4A,B). MMR, AS, Ucrit, and COT, but not SMR, were positively affected by higher dietary iron levels following 20 days of exposure (Figures 4C–F; Supplementary Figure S3). However, by Day 40, some of these positive effects appeared to diminish. At that time point, fish fed the Low and Medium Fe diets showed increases in Ucrit and MMR, respectively.

There were no statistically significant differences in glycogen or triglyceride levels among zebrafish fed Low, Medium, or High Fe diets (Table 2). However, within the Low Fe group, glycogen levels appeared elevated at Day 40, and triglyceride levels appeared elevated at Day 20.

**TABLE 2:** Glycogen and triglyceride concentrations (mg/g) in the carcass of adult zebrafish exposed to different dietary iron treatments. Data are mean ± SEM; n = 3–4. No statistical difference noted following one-way ANOVA, p < 0.05.

Diet	Glycogen (mg/g)		Triglyceride (mg/g)	
	Day 20	Day 40	Day 20	Day 40
Low Fe	3.99 ± 0.9	9.46 ± 3	17.8 ± 4	10.9 ± 2
Medium Fe	5.15 ± 3	3.40 ± 0.7	8.57 ± 1	9.89 ± 3
High Fe	3.82 ± 0.8	4.99 ± 1	7.93 ± 3	13.0 ± 2

### 3.4 Reproductive performance

**FIGURE 5:**
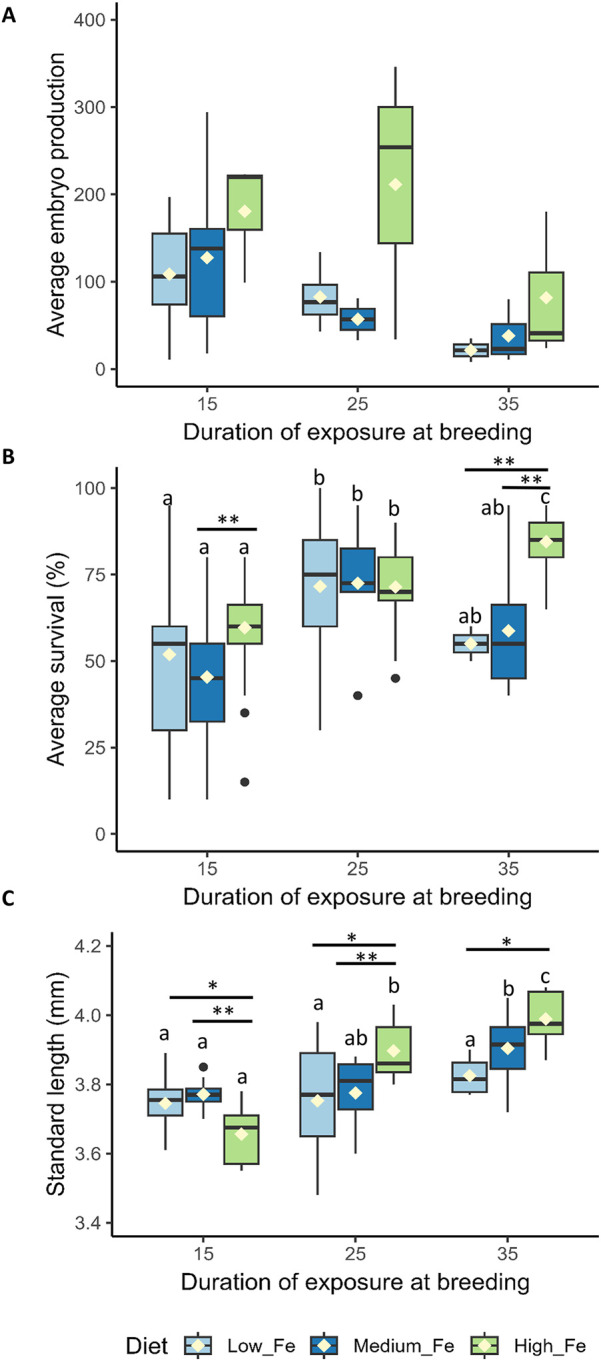
Reproductive performance and offspring quality in zebrafish following parental dietary iron exposure. **(A)** Average embryo production per breeding pair (n = two to seven successful breeding pairs per session). **(B)** Percentage embryo survival 1 day post-fertilization (n = 28–29 replicates for Day 15, n = 6–32 for Day 25, and n = 3–12 for Day 35 per dietary treatment; each replicate consists of 20 embryos). **(C)** Standard length (SL) of larvae at 5 days post-fertilization, derived from parents exposed to Low Fe, Medium Fe, or High Fe diets for 15, 25, or 35 days. Asterisks indicate significant (^*^ *p* < 0.05, ^**^ *p* < 0.01) differences among dietary iron treatments within an exposure period; lowercase letters indicate significant differences between exposure periods within the same dietary iron treatment. Statistical analysis for average embryo production and percentage embryo survival was performed using a permutation-based two-way ANOVA (10,000 iterations), followed by pairwise permutation tests (FDR-adjusted, *p* < 0.05) to compare group differences. Two-way ANOVA followed by a *post hoc* Holm-Sidak test was used to compare SL between treatment groups; *p* < 0.05 (n = 4–10 fish per treatment).

Adult fish exposed to Low, Medium, and High dietary iron treatments were bred every 10 days to assess fertility. Of the eight breeding pairs (1 male:1 female) per treatment group, two to seven pairs successfully bred during each session (Figure 5A). Embryo production per breeding pair varied considerably, with no significant differences among treatment groups (p > 0.05). However, the Medium Fe group showed the highest breeding success at Day 15, with 7 of 8 pairs producing embryos. With repeated breeding, the number of successful breeding pairs decreased on Day 35 (from seven to three at Day 15 to two to three at Day 35). Although embryo production varied widely between breeding sessions with no statistical differences, the High Fe group produced the most cumulative embryos (total embryos collected on Days 15, 25, and 35) (Supplementary Figure S4). This group produced approximately 300 more embryos than the Medium Fe group and 500 more than the Low Fe group.

Embryos from parents exposed to 35 days of High Fe exhibited the highest survival rate compared to the other treatment groups (*p* < 0.01) (Figure 5B). No significant differences in survival were observed in embryos collected from parents exposed to 15 and 25 days of Fe.

Offspring of parent fish exposed to 15 days of High Fe treatment had the lowest SL (Figure 5C). In contrast, longer parental High Fe exposure led to significant increases in larval SL. An increase in SL was also observed in the Medium Fe group following 35 days of parental iron exposure. The duration of Low Fe exposure did not affect the SL of the offspring.

**FIGURE 6:**
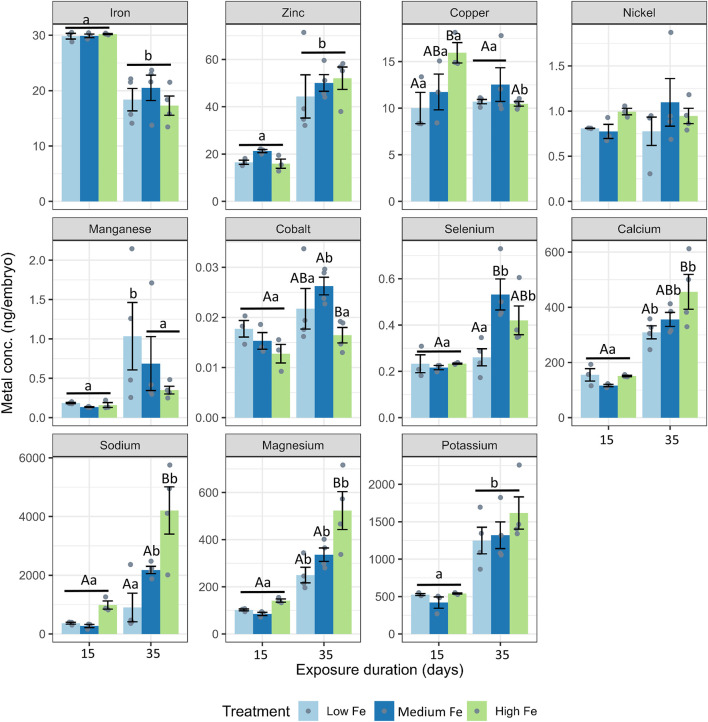
Elemental composition of zebrafish embryos derived from parents exposed to dietary iron treatments. Concentrations of iron, zinc, copper, nickel, manganese, cobalt, selenium, calcium, sodium, magnesium, and potassium (ng/embryo) in embryos collected at 15 and 35 days of parental exposure to Low Fe, Medium Fe, and High Fe diets. Data are mean ± SEM, n = 3–4 (each replicate consisted of a pooled sample from 10–20 fish). Two-way ANOVA followed by a *post hoc* Holm-Sidak test, *p* < 0.05. Uppercase letters indicate significant differences among dietary iron treatments within an exposure duration; lowercase letters indicate differences between exposure durations within the same dietary treatment.

During the Day 15 and Day 35 breeding sessions, embryos were collected for trace metal and major ion analysis (Figure 6; Supplementary Figure S5). Iron was the most abundant trace metal in embryos from the Day 15 parental exposure group. Interestingly, zinc appeared to be more abundant in embryos in the Day 35 exposure group. Also, at Day 15, copper levels in embryos from High Fe treated parents were significantly higher than those from Low Fe groups. In embryos from the Day 35 exposure group, major ions such as calcium, magnesium, and sodium were significantly higher in the High Fe group compared to the Low Fe group. Apart from selenium at Day 35, all other metals and ions measured were not different between the Low and Medium Fe groups.

### 3.5 Physiological condition, swimming performance, and metabolic rate of the offspring

#### 3.5.1 Physiological condition

Embryos from the parental iron treatment groups were raised to adulthood and were treated with the same dietary iron regimen as their parents. There were no significant differences in weight, SL, or condition factor among the three offspring groups following dietary Fe exposure (Table 3). Condition factor was also comparable between the offspring groups and their respective parent groups at Day 20 (Table 1).

**TABLE 3:** Body weight (g), standard body length (cm), and condition factor (g/cm^3^) of male and female offspring exposed for 20 days to the same dietary iron treatments (Low, Medium, and High Fe) as their parents. Data are mean ± SEM; n = 10–13. No statistical difference noted following one-way ANOVA, p < 0.05.

Diet	Body weight (g)	Standard body length (cm)	Condition factor (g/cm^3^)
Low Fe	0.69 ± 0.05	3.3 ± 0.1	1.8 ± 0.1
Medium Fe	0.54 ± 0.02	3.2 ± 0.0	1.7 ± 0.0
High Fe	0.63 ± 0.04	3.3 ± 0.1	1.7 ± 0.1

#### 3.5.2 Swim and metabolic parameters

**FIGURE 7:**
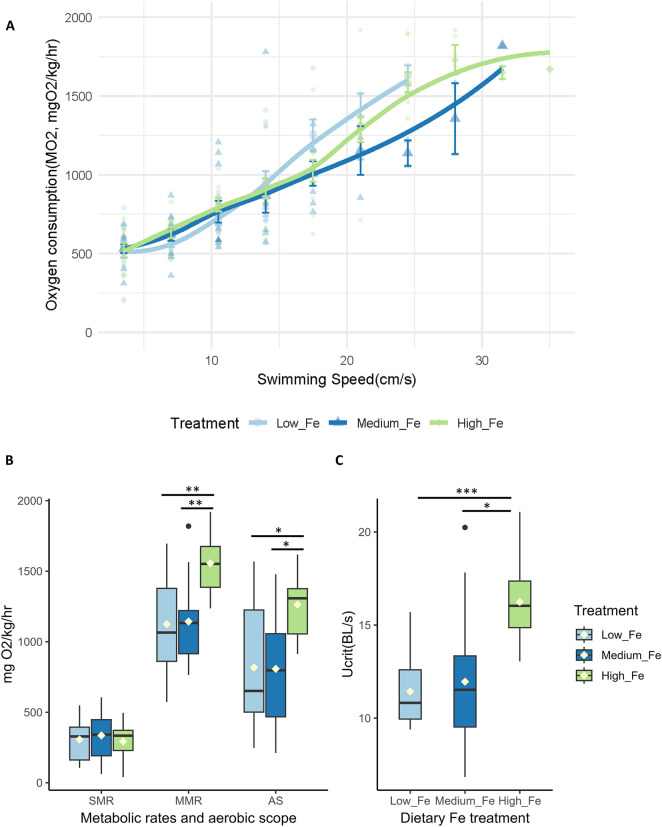
Intergenerational effects of parental dietary iron exposure on swimming performance and metabolism in offspring. **(A)** Oxygen consumption rates (MO_2_, mgO_2_/kg/hr) during a critical swim test in adult offspring (F_1_) exposed for 20 days to the same dietary iron treatment as their parents (Low Fe, Medium Fe, or High Fe). Each data point represents an individual measurement (n = 12–13 fish at starting speed and n = 1-2 fish at ending speed), with different symbols/colors indicating different experimental groups. Non-linear regression lines were fitted using locally estimated scatterplot smoothing (LOESS) to illustrate trends in metabolic demand across swimming speeds. Statistical differences were assessed using a two-way repeated-measures ANOVA, followed by *post hoc* Holm-Sidak test, *p* < 0.05. **(B)** Standard metabolic rate (SMR), maximum metabolic rate (MMR), and aerobic scope (AS) (n = 11–12 fish per treatment group). **(C)** Critical swimming speed (body lengths/s; n = 11–12 fish per treatment group). Asterisks denote statistical significance (^*^ *p* < 0.05, ^**^ *p* < 0.01, ^***^ *p* < 0.001) among dietary iron groups; (One-way ANOVA; *p* < 0.05).

Offspring exposed to Low, Medium, or High Fe diets for 20 days exhibited similar trends in swimming and metabolic performance as their parents at Day 20 (Figure 7; Supplementary Figure S6). Offspring from the High Fe group showed significant improvement in swimming performance, with maximum sustained swimming speeds reaching 35 cm/s in High Fe offspring, compared to 24.5 cm/s in Low Fe fish (Figure 7A). Metabolic parameters including MMR, AS, and Ucrit were also elevated in the High Fe group (Figures 7B,C).

**FIGURE 8:**
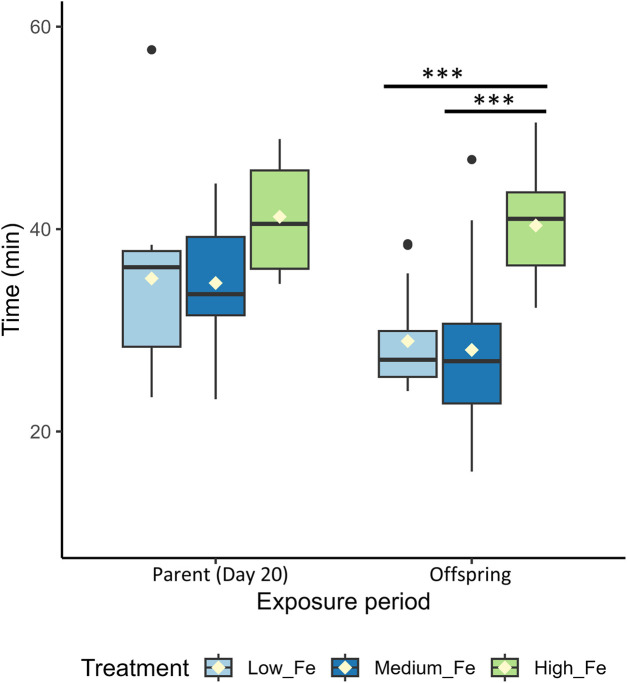
Endurance performance in parent and offspring zebrafish following dietary iron exposure. Time (min) to fatigue during a critical swim test in adult zebrafish exposed to dietary iron treatments (Low Fe, Medium Fe, and High Fe). Groups include Parent (Day 20) and Offspring (exposed for 20 days to the same dietary treatment as their parents). Asterisks indicate significant differences (^***^ *p* < 0.001) among dietary iron treatments within an exposure period (One-way ANOVA; *p* < 0.05, n = 7–13 per treatment group).

The difference in Ucrit between Low and High Fe groups was most pronounced in the offspring (*p* < 0.001), exceeding the disparity observed in Day 20 (*p* < 0.01) and Day 40 (*p* > 0.05) parents (Figure 7; Figure 4). A similar pattern was observed for time to fatigue, which differed significantly between Low and High Fe offspring (*p* < 0.001) but not in parents (Figure 8). The offspring group displayed the largest increases in MMR, Ucrit, and fatigue time between Low and High Fe treatments when compared with their parents.

## 4 Discussion

### 4.1 Overview and physiological responses of adult zebrafish to dietary iron levels

Fish need to tightly regulate iron balance to meet metabolic requirements while also avoiding excessive accumulation under changing dietary iron availability. In the present study, zebrafish were fed diets representing iron deficiency (11 mg Fe/kg), adequate/control levels (420 mg Fe/kg), and iron-enriched (2,300 mg Fe/kg), to reflect environmentally relevant differences in dietary iron availability. Although precise dietary requirements for zebrafish remain undefined, the three diets represented a gradient from below sufficiency to excess. The Low Fe diet contained approximately three times less iron than the lowest recommended dietary level reported for fish (Andersen et al., 1996). Here, we observed that there was no significant difference in weight, body length, condition factor, and HSI as a result of dietary iron concentrations. Our data indicate that zebrafish tolerated sub-chronic exposure to low dietary Fe without major physiological deficits, which is possibly buffered by pre-existing iron reserves. While the acclimation period (7 days) to the Medium Fe diet may have been insufficient to stabilize iron reserves in the Low Fe group, it is likely that the pre-exposure diets (∼300 mg Fe/kg daily during regular rearing) could have facilitated the accumulation of sufficient iron reserves for the duration of this experiment. In a similar study, no adverse effects were reported in zebrafish fed a diet containing 33 mg Fe/kg, although a decline in body weight became apparent after 10 weeks of exposure (Cooper et al., 2006).

Despite no difference among dietary groups, condition factor, HSI, and GSI were generally higher at Day 20 than Day 40. These changes coincided with the initial differences in body weight between Day 20 and Day 40 fish groups, leading to the higher physiological indices observed within the Day 20 group. The variation between Day 20 and 40 was further magnified by reproductive demands such as repeated spawning, reflected in the significantly lower GSI observed at Day 40. In particular, female zebrafish can release hundreds of eggs, which may have also contributed to weight fluctuations during the trial. A decrease in GSI of up to 5% has been reported in females following consecutive breeding sessions (Hoo et al., 2016). Nonetheless, all parameters remained within ranges that were considered normal, and there were no other overt changes in their feeding activity (Frederickson et al., 2021). Only the females from the Medium Fe group showed no change in physiological metrics throughout the exposure duration. The iron level in this Medium Fe diet was the most similar to their pre-exposure conditions and was likely the most nutritionally optimal of the three experimental diets for maintaining all physiological indices. Furthermore, there were no apparent benefits of high iron supplementation to general physiological conditions, and both the condition factor and HSI were decreased in both sexes within this group during prolonged exposure.

### 4.2 Tissue burden of trace metals and major ions

Dietary iron is absorbed in the intestine and distributed throughout the body for storage and metabolic use. Iron and its transport pathways are known to interact with multiple other metals, including zinc, copper, nickel, and calcium, and dietary iron exposure has been shown to modulate the expression of zinc (Zrt- and Irt-like protein; *zip8* and *zip14*) and copper (copper transport 1; *ctr1*) transporters, and calcium (epithelial calcium channel; *ecac*) channel (Gunshin et al., 1997; Qiu and Hogstrand, 2004; Kwong and Niyogi, 2009; Chandrapalan and Kwong, 2020). In particular, the highly conserved major iron transporter, divalent metal transporter 1 (DMT1), was found to have broad specificity to multiple divalent metals (iron, zinc, manganese, cobalt, cadmium, copper, nickel, and lead) in mammals (Gunshin et al., 1997; Garrick et al., 2003). These studies suggest potential interactions among metal uptake mechanisms and multi-metal homeostasis. In the present study, we found that dietary iron altered tissue metal burdens, most notably increasing iron in the intestine and brain. While the deleterious effects of iron in the intestine are less understood, the accumulation in the brain may have functional implications, consistent with earlier reports of neurobehavioral sensitivity to iron (Hassan and Kwong, 2020). Although we observed tissue iron accumulation, we did not directly measure biomarkers of oxidative stress (e.g., lipid peroxidation, ROS production, antioxidant enzyme activity) or tissue-level pathology, which would be important indicators of iron toxicity (Pereira et al., 2016; Chowdhury and Saikia, 2022; Formicki et al., 2025). While iron toxicity is not the scope of this study, future work incorporating these endpoints would be useful to understand whether the elevated iron burdens translate into cellular or organ-level damage. Nonetheless, among all organs examined, the intestine was the most affected by dietary Fe treatment, with almost all measured metals/ions (excluding calcium) showing significant changes either across exposure duration or between dietary treatment. Notably, there did not appear to be a consistent trend, likely reflecting complex metal–metal interactions in the intestine via DMT1 and other shared metal transport pathways (e.g., ZIPs and ECaC) (Pinilla-Tenas et al., 2011; Kondaiah et al., 2019; Chandrapalan and Kwong, 2020).

Furthermore, fish exposed to the Low Fe treatment maintained iron levels comparable to the Medium Fe group. It is possible that the Low Fe diet with deficient iron content, along with pre-existing iron reserves, was sufficient to mitigate iron dysregulation in these zebrafish.

### 4.3 Swimming capacity and energy metabolism

We demonstrated the nutritional benefits of sub-acute iron supplementation, as fish showed enhanced capacity for sustained swimming and exhibited higher metabolic rates. Fish fed the High Fe diet had significantly higher Ucrit (∼1.5x of the Medium Fe group), surpassing the reported average for wildtype zebrafish (∼12.5 BL/s) (Plaut, 2000). These results provide evidence that iron supplementation enhances both aerobic scope and endurance capacity and may better meet the higher energetic demands during sustained swimming activities. Despite improvements in swimming performance, there were no changes in glycogen and triglyceride (primary energy sources for swimming) levels among the three dietary Fe groups. Notably, elevated ATP and hemoglobin levels were previously reported in iron-treated masu salmon (Mizuno et al., 2007), and incorporating similar measures into zebrafish studies would be valuable going forward. Since iron levels undermine ATP and hemoglobin production, iron supplementation and corresponding increases in body iron reserves may help to sustain the increase in energy and oxygen transport demands associated with enhanced swimming performance. Indeed, fish experiencing anemia and hypoxia display reduced swimming performance from limited oxygen carrying capacity and aerobic metabolic scope (Wagner and McKinley, 2004; Domenici et al., 2013).

The present study also showed signs of a potential plateau in swimming performance following sub-chronic supplementation with High Fe. Reductions in swimming performance are commonly observed in fish inhabiting metal-contaminated environments or experiencing persisting metal-induced stress (van der Oost et al., 2020; Gashkina, 2024). Previous studies also found that sublethal exposure to metals or metalloids disrupted metabolic enzymes and increased cortisol levels, impairing energy homeostasis (Handy et al., 1999; Almeida et al., 2001; Thomas and Janz, 2011; Javed and Usmani, 2015; Pettem et al., 2017). Similarly, masu salmon supplemented with 1500 but not 3,000 mg Fe/kg for 3 months improved sprint swimming ability (Mizuno et al., 2007). Together, these results suggest a dual effect where sub-acute iron supplementation can enhance swimming capacity and metabolism, but prolonged exposure may lead to toxicity, highlighting the narrow balance between benefit and toxicity. During prolonged exposure to high levels of iron, a threshold may exist where supplementation becomes metal stress. Fish in these circumstances require metabolic trade-offs, often diverting their energy toward detoxification (e.g., hepatic clearance) and reestablishing homeostasis, thereby limiting the energy available for other activities, including locomotion (Handy et al., 1999). Since sub-chronic exposure to High Fe was also associated with iron accumulation in organs like the brain and intestine, possible toxicity may underlie observed declines in swimming performance over time. It is also important to acknowledge the potential of repeated handling stress on swimming performance during concurrent breeding trials. As such, measuring biomarkers of oxidative stress (as mentioned above) and cortisol could help understand whether these effects lead to functional impairments in swimming performance (Clark et al., 2011; Formicki et al., 2025).

### 4.4 Reproductive output and early development

Our study showed that dietary iron supplementation improved reproductive output, embryo survival, and early larval growth. Zebrafish fed the High Fe diet consistently produced more embryos across all three breeding events. Zebrafish are known for their high fecundity, and within the course of the 20-day breeding period, they produced 900–1400 embryos. However, the reproductive output in zebrafish can be inherently variable, both among individuals and across breeding sessions. This variability is influenced by factors such as age, spawning frequency, and environmental conditions. Although our statistical analyses accounted for treatment effects, such variability may mask subtle differences among dietary groups. Future work should consider larger sample sizes or mixed-effect models that better accommodate repeated breeding events and individual-level variation (Hoo et al., 2016; Wafer et al., 2016). The total number of eggs produced per session also generally declined over time, possibly from the stress of repeated breeding sessions. As oviparous fish, zebrafish embryos also rely on maternally supplied yolk nutrients (Reading et al., 2018). Interestingly, trace metal content in embryos appeared to vary inversely with the number of eggs produced at the time of breeding. Embryos from the final breeding event (Day 35) appeared to have higher metal content except for iron, copper, and nickel, but this session also produced the fewest eggs. Although these effects were non-specific to dietary treatment, they were most likely reflecting maternal depletion or selective allocation of nutrients. Previous studies have reported that maternal metal/metalloid exposure can influence offspring metal/metalloid content, including the transfer of zinc and selenium (Beaver et al., 2017; Thomas and Janz, 2015). In contrast, our findings suggested that parental iron exposure did not lead to increased iron content in the embryos. Interestingly, in embryos collected on Day 35, we observed a reduction in manganese content, but increases in calcium, sodium, and magnesium contents with increasing parental dietary Fe exposure. It seems that parental iron status may influence the deposition of certain trace metals and major ions in embryos.

### 4.5 Offspring physiological responses and swimming performance

Previous studies of parental exposure to waterborne metals have primarily demonstrated intergenerational effects through altered embryonic metal loads or disrupted developmental processes. For example, early life exposure to zinc was found to disrupt metal homeostasis in adult zebrafish and their offspring, with persistent alterations in genes involved in metal regulation (Zheng et al., 2022). Similarly, parental copper exposure has been linked to altered developmental processes in the next-generation through epigenetic mechanisms (Tai et al., 2022). One of the most compelling findings of our study is the observation that not only waterborne exposures, but dietary exposure to iron can also elicit intergenerational effects on offspring performance. In particular, offspring of the sub-acute High Fe parents exhibited significant improvements in aerobic scope and swimming performance, traits that are closely linked to ecological fitness. Importantly, the differences in Ucrit and fatigue resistance between the Low and High Fe groups were even more pronounced in the offspring generation. The exact mechanism underlying these intergenerational effects would be an important area to address in future studies, particularly to identify whether the enhanced offspring performance results from direct maternal iron transfer to gametes and/or epigenetic reprogramming. While we did not observe differences in iron content among the embryos from the three dietary iron groups, recent advances in epigenetic research suggested that exposure to environmental stressors can induce changes in DNA methylation, histone modification, and small RNA expression, leading to persisting transgenerational effects (Cecere, 2021; Pierron et al., 2022; Pham et al., 2023; Abdelnour et al., 2024). Incorporating such epigenetic analyses in future studies on iron exposure could provide mechanistic insight into any observed intergenerational effects.

Nonetheless, the intergenerational physiological adjustment to repeated dietary iron exposure highlights the importance of parental iron status and its lasting effects on offspring fitness. These findings reveal that dietary iron not only shapes immediate metabolic and performance outcomes in adults but can also program offspring traits with potential ecological consequences. In natural ecosystems, fluctuations in dietary iron availability, driven by environmental stressors such as hypoxia, acidification, or mining runoff, may have the potential to alter predatory-prey dynamics, foraging efficiency, and reproductive success by influencing both parental condition and offspring swimming capacity. By demonstrating that dietary iron has potential intergenerational effects on traits directly tied to survival and fitness, our study highlights the complex ecological consequences of dietary iron availability.

### 4.6 Conclusions

The present study reveals a complex relationship between dietary iron exposure, metal homeostasis, and swimming performance in zebrafish. We found that zebrafish tolerated both sub-acute and sub-chronic exposure to deficient and high levels of dietary iron without apparent detriments to general health; however, tissue-specific iron accumulation was observed in the High Fe treatment group following prolonged exposure. Notably, a 20-day exposure to high dietary iron led to enhanced critical swimming speed, aerobic scope, and maximum metabolic rate, as well as improved endurance capacity. These performance improvements were even more pronounced in the offspring, underscoring the potential intergenerational effects of dietary iron exposure. Together, our findings demonstrate that dietary iron availability can shape both parental and offspring performance.

From an ecological perspective, our results highlight the importance of iron levels in freshwater habitats, where natural fluctuations and anthropogenic activities can influence iron concentrations and potentially affect the fitness of wild fish populations. Additionally, understanding how parental iron nutrition impacts offspring performance could inform aquaculture practices, where optimizing feed formulations may enhance survival, growth, and resilience in subsequent generations. Future studies should focus on elucidating the molecular and physiological mechanisms underlying these intergenerational effects, with particular emphasis on iron and energy metabolism, as well as the potential roles of epigenetic modifications, transporter regulation, and interactions with other trace metals.

## Data Availability

The raw data supporting the conclusions of this article will be made available by the authors, without undue reservation.
