# Supplementary material for: Intergenerational effects of dietary iron on swimming and metabolic performance in zebrafish
Source: Front Physiol. 2025 Oct 16;16:1693900. doi: 10.3389/fphys.2025.1693900 (PMC12571662; doi:10.3389/fphys.2025.1693900)
Supplement: Supplementary file 2 [file Table1.docx]

**Supplementary Tables**

**Table S1. Nutritional composition of purified zebrafish diet deficient in Fe used to prepare the experimental diets.**

| **Ingredient** | **Concentration** (**g/Kg**) |
| --- | --- |
| Cornstarch | 329 |
| Dyetrose* | 110 |
| Microcrystalline Cellulose | 190 |
| Soybean Oil | 30.0 |
| Menhaden Oil | 30.0 |
| Ethoxyquin (0.1%) | 0.060 |
| L-Amino Acid mix | 239 |
| Salt Mix #295004 (Rx, no Fe) | 40.0 |
| Sodium Bicarbonate | 14.3 |
| Vitamin Mix #390015 | 10.0 |
| L-Methionine | 6.12 |
| Choline Chloride | 1.67 |

* Depolymerized cornstarch which aids in the pelleting process.

**Table S2.** **Measured Fe concentration (mg Fe/kg) in experimental diets and the daily dose of Fe (µg Fe/g fish wt/day).** Data are mean ± SEM; n = 3.

| **Dietary treatment** | **Fe concentration** | **Daily Fe dose** |
| --- | --- | --- |
| Low Fe | 11 ± 0.8 | 0.17 |
| Medium Fe | 420 ± 50 | 6.3 |
| High Fe | 2300 ± 80 | 35 |

**Table S3.** **Elemental composition (mg/kg) in the purified iron deficient diet purchased from Dyets Inc. and the experimental diets (Low Fe, Medium Fe, and High Fe).** Data are mean ± SEM; n = 4 for Purified base diet, and n =9 for Experimental diets.

| **Element** | **Purified base diet** | **Experimental diets** |
| --- | --- | --- |
| Zn | 48 ± 2 | 61 ± 5 |
| Cu | 36 ± 9 | 34 ± 7 |
| Ni | 0.42 ± 0.01 | 0.29 ± 0.06 |
| Mn | 67 ± 20 | 66 ± 2 |
| Co | 0.24 ± 0.02 | 0.31 ± 0.01 |
| Se | 0.052 ± 0.002 | 0.08 ± 0.0048 |
| Ca | 7200 ± 200 | 6800 ± 200 |
| Na | 5500 ± 70 | 5500 ± 270 |
| Mg | 190 ± 40 | 310 ± 8 |
| K | 3800 ± 500 | 4000 ± 200 |

**Table S4.** Weight (g) of male and female zebrafish exposed to Low, Medium, and High Fe diet for a duration of 20 and 40 days. Data are mean ± SEM, n=9-13 (per sex, per treatment). One-way ANOVA, *p* < 0.05. Lowercase letters denote statistical significance between pre- and post-exposure within timepoint and within sex.

| **Day** | **Sex** | **Pre-exposure** | **Post-exposure** | | |
| --- | --- | --- | --- | --- | --- |
|  |  |  | Low Fe | Medium Fe | High Fe |
| 20 | Male | 0.62±0.02^a^ | 0.54±0.03^ab^ | 0.51±0.02^b^ | 0.52±0.02^b^ |
|  | Female | 0.74±0.02^a^ | 0.64±0.02^b^ | 0.59±0.03^b^ | 0.60±0.02^b^ |
| 40 | Male | 0.48±0.02^a^ | 0.48±0.03^a^ | 0.45±0.02^a^ | 0.46±0.02^a^ |
|  | Female | 0.54±0.03^a^ | 0.53±0.02^a^ | 0.51±0.02^a^ | 0.55±0.02^a^ |
